# Accidental Interaction between PDZ Domains and Diclofenac Revealed by NMR-Assisted Virtual Screening

**DOI:** 10.3390/molecules18089567

**Published:** 2013-08-09

**Authors:** Takeshi Tenno, Natsuko Goda, Yoshitaka Umetsu, Motonori Ota, Kengo Kinoshita, Hidekazu Hiroaki

**Affiliations:** 1 Laboratory of Structural Molecular Pharmacology, Graduate School of Pharmaceutical Sciences, Nagoya University, Furocho, Chikusa-ku, Nagoya 464-8601, Japan; E-Mails: tenno.takeshi@e.mbox.nagoya-u.ac.jp (T.T.); tenno.natsuko@f.mbox.nagoya-u.ac.jp (N.G.); 2 Division of Structural Biology, Graduate School of Medicine, Kobe University, 7-5-1 Kusunoki-cho, Chuo-ku, Kobe, Hyogo 650-0017, Japan; E-Mail: umetsu@jaist.ac.jp; 3 Center for Nano Materials and Technology, Japan Advanced Institute of Science and Technology, 1-1 Asahidai, Nomi, Ishikawa 923-1292, Japan; 4 Department of Complex Systems Science, Graduate School of Information Sciences, Nagoya University, Nagoya 464-8601, Japan; E-Mail: mota@is.nagoya-u.ac.jp; 5 Department of Applied Information Sciences, Graduate School of Information Sciences, Tohoku University, Miyagi 980-8597, Japan; E-Mail: kengo@ecei.tohoku.ac.jp; 6 The Structural Biology Research Center and Division of Biological Science, Graduate School of Science, Nagoya University, Furo-cho, Nagoya 464-8601, Japan

**Keywords:** PDZ domains, protein–protein interaction inhibitor, non-steroidal anti-inflammatory drug, drug repositioning

## Abstract

*In silico* approaches have become indispensable for drug discovery as well as drug repositioning and adverse effect prediction. We have developed the eF-seek program to predict protein–ligand interactions based on the surface structure of proteins using a clique search algorithm. We have also developed a special protein structure prediction pipeline and accumulated predicted 3D models in the Structural Atlas of the Human Genome (SAHG) database. Using this database, genome-wide prediction of non-peptide ligands for proteins in the human genome was performed, and a subset of predicted interactions including 14 PDZ domains was then confirmed by NMR titration. Surprisingly, diclofenac, a non-steroidal anti-inflammatory drug, was found to be a non-peptide PDZ domain ligand, which bound to 5 of 15 tested PDZ domains. The critical residues for the PDZ–diclofenac interaction were also determined. Pharmacological implications of the accidental PDZ–diclofenac interaction are further discussed.

## 1. Introduction

Protein–ligand docking, a virtual *in silico* screening approach, is an indispensable technology for drug discovery. Many protein–ligand docking programs have been developed and are widely used [1,2,3]. Both the commercial applications such as Glide [4], MOE/ASEDock [5], GOLD [6], FLOG [7], and FRED [8], and the academic applications, such as AutoDock [9] and Sievgene [10], are useful. Recently, such *in silico* approaches have also been utilized for drug repositioning [11,12,13] and adverse effect prediction [14,15]. In all cases, fast and accurate methods need to be further developed.

In our previous studies, we developed a method called eF-seek [16] to predict ligand binding sites in a new protein structure by searching for similar binding sites that were already listed in the Protein Data Bank (PDB). eF-seek locates potential ligand binding sites in a protein structure using a clique search algorithm; if similar structures were deposited in the eF-site, the database searches for ligand binding sites [17,18]. This tool was initially developed for annotating biochemical functions of proteins based on 3D protein structures. Later, the tool was included in the pipeline for automatic annotation of all human genome products with fully automated 3D structure prediction, which are summarized in the SAHG database [19]. Since eF-seek is sensitive to input of 3D coordinates, the application of the program through the pipeline worked well only when highly accurate structure models were provided, *i.e*., the templates were detected by BLAST search with as high as 90% sequence identity. Therefore, experimental confirmation of the newly found protein–ligand pairs annotated in the SAHG database was initiated. Consequently, a set of protein–ligand pairs was selected by focusing on PDZ domains in the human genome.

PDZ (PSD95/Discs large/ZO-1) domains are highly conserved compact globular modules of ~90 residues. They typically recognize specific C-terminal motifs and, in doing so, assemble multicomponent protein complexes inside eukaryotic cells [20,21]. As one of the most abundant cytosolic protein modules in the human genome, there may be as many as 440 PDZ domains in 259 different proteins [22]. Several PDZ domain-containing proteins (PDZ proteins) are known to be related to diseases, such as cancer [23,24,25], Parkinson’s disease [26], cerebral ischemia [27], and Alzheimer’s disease [28]. As a result, PDZ domains have been considered attractive candidates for drug discovery [29]. Recently, some non-peptide inhibitors of PDZ domains have emerged [30,31,32]. Thus, we focused on the predicted protein–ligand sets containing PDZ domains in the SAHG database.

In this study, we employed a high-throughput construction method for protein expression vectors in *Escherichia coli*, called the PRESAT-vector system [33], to construct as many as 14 human PDZ domains. We also employed NMR titration to confirm whether any of the 10 predicted ligands could bind to the target PDZ domains. Finally, we discovered that diclofenac and flufenamic acid, which are well-known non-steroidal anti-inflammatory drugs (NSAIDs), accidentally bound to several (5 of 15 tested) PDZ domains.

## 2. Results and Discussion

### 2.1. eF-Seek Search for Non-peptide Ligands of PDZ Domains in the Human Genome

The SAHG database (http://bird.cbrc.jp/sahg) contains highly accurate homology models of many human genome proteins. Among the database, 956 accurate protein models derived from 10 chromosomes were selected as the initial eF-seek screening targets. Among them, 709 models gave prediction results with a positive threshold higher than 50%. An average of three ligands per protein was predicted (2,095 ligand–protein pairs). 

### 2.2. Target Selection

We then limited the number of the target protein–ligand pairs according to the following four issues: (1) the target protein should be readily expressed in an *E. coli* system; (2) the predicted ligands should be drug-like compounds; (3) the predicted ligands should possess different skeletal structures than their natural ligand counterparts; and (4) the predicted ligands should be able to inhibit any interaction of the target proteins. Based on these criteria, 114 domains were listed. The domains included 28 RNA binding domains, 27 ubiquitin-like/ubiquitin-related domains, 17 PDZ domains, 11 SH3 domains, five DEATH and PH domains, and 23 others. Simultaneously, 351 protein–ligand pairs and 85 individual ligands were assessed.

Then, we focused on PDZ domains, as they play key roles in post synaptic density and neural membrane protein signaling. The predicted 17 PDZ domains gave 23 ligands. Among 17 PDZ domains, we succeeded in constructing 14 PDZ domain expression vectors in the form of a GST fusion protein. We also added another PDZ domain, mouse ZO1-PDZ1, as a control. Among 23 compounds, 13 were readily available commercially; however, three were insoluble in either H_2_O or DMSO. The list of 14 + 1 PDZ domains is shown in Table 1. The list of the 10 compounds examined in this study is shown in Table 2. Although most of PDZ domains are soluble and well expressed in *E. coli*, three PDZ domains (PDZ1, PDZ3, and PDZ14) did not give soluble recombinant protein samples (denoted as * in Table 1).

### 2.3. NMR Titration Experiments of PDZ Domains with the Predicted Ligands

**Figure 1:**
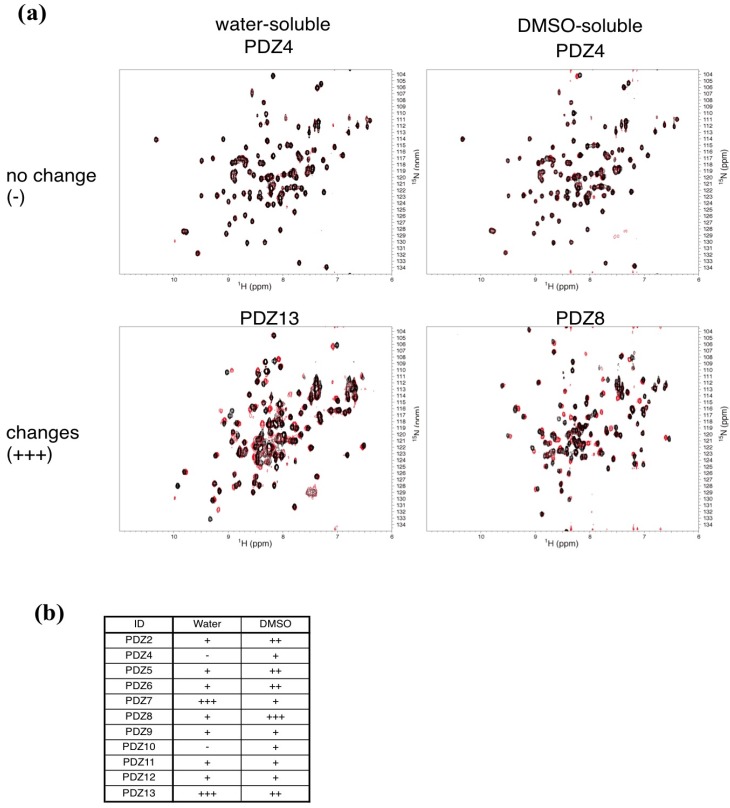
Examples of NMR-binding assay between PDZ domains and the compound cocktails. (**a**) Each overlaid spectrum was derived from a PDZ domain with (red) and without (black) cocktail. Upper spectra show that no signal changes were observed with mixing of the PDZ domains and a cocktail. Lower spectra show the signal changes when PDZ domains were mixed with a cocktail. (**b**) Summary table of binding assays using compound cocktails. The number of the plus signs indicates the degree of signal changes (+, less than 15 signals changed; ++, less than 30; +++, over 30, respectively). The minus sign indicates no signal changes. PDZ1, PDZ3, and PDZ14 were not examined.

In our preliminary study, we failed to detect any PDZ–ligand interaction with reasonable signal-to-noise ratio using WaterLOGSY methods [34]. Thus, we switched to a “protein-based” NMR approach rather than the “ligand-based” approach for the experimental confirmation of PDZ–ligand interactions [35]. Subsequently, we prepared PDZ domain samples as ^15^N-labeled forms, and a series of 2D-NMR-based experiments was performed. Since we used very low concentrations of PDZ domain samples (25 μM), the use of SOFAST-HMQC technique, which provides a spectrum of a high signal-to-noise ratio within a reasonable measurement period [36], is absolutely necessary. At the first stage of the experiments, the 10 compounds were divided into two cocktails, group I (containing six water-soluble compounds) and group II (containing four DMSO-soluble compounds). Subsequently, these two cocktails were added to each of the 11 PDZ domains, and each of the HMQC spectra with and without drugs were compared. We observed significant chemical shift changes in PDZ7, PDZ8, and PDZ13 (Figure 1). In addition, PDZ5, PDZ6, PDZ9, and PDZ11 showed intermediate changes.

**Table 1:** PDZ domains used in this study.

ID	Sample name	RefSeq ID	Length
PDZ1 *	NHE-RF1_PDZ1	NP_004243	88
**PDZ2**	LNX2_PDZ2	NP_699202	89
PDZ3 *	NHE-RF4_PDZ3	NP_079067	93
**PDZ4**	Stxbp4_PDZ1	NP_848604	95
**PDZ5**	DVL2_PDZ	NP_004413	98
**PDZ6**	DVL1_PDZ	NP_004412	98
**PDZ7**	RHPN2_PDZ	NP_149094	99
**PDZ8**	Harmonin_PDZ2	NP_710142	100
**PDZ9**	InaDL_PDZ8	NP_795352	106
**PDZ10**	LIMK2_PDZ	NP_057952	107
**PDZ11**	Harmonin_PDZ3	NP_710142	108
**PDZ12**	Neurabin-2_PDZ	NP_115984	110
**PDZ13**	InaDL_PDZ6	NP_795352	113
PDZ14 *	PAR-6beta_PDZ	NP_115910	127
**mZO-1 PDZ1**	Mouse ZO-1_PDZ1	NP_009386	94

**Table 2:** Small compounds used in this study.

Water-soluble			DMSO-soluble		
ID	Name	Structural formula	ID	Name	Structural formula
A2G	*N*-acetyl-2-deoxy-2-amino-galactose	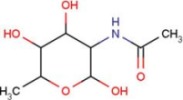	MPB	4-hydroxy-benzoic acid methyl ester	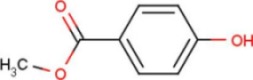
DIF	2-[(2,6-dichlorophenyl) amino] benzene-acetic acid	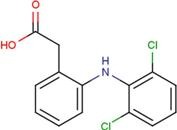	FLF	2-[[3-(trifluoro-methyl) phenyl] amino] benzoic acid	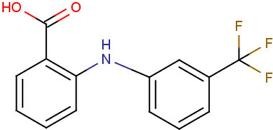
FUA	fusidic acid	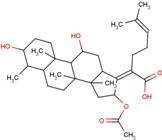	MIL	milrinone	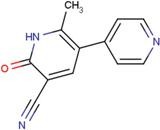
SIA	*O*-sialic acid	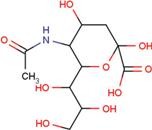	MNA	2-*O*-methyl-5-*N*-acetyl-α-D-neuraminic acid	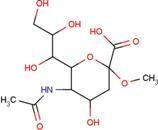
NES	2-(2-hydroxy-1,1-dihydroxymethyl-ethylamino)-ethanesulfonic acid	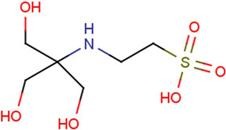	TRP	tryptophan	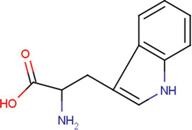

**Figure 2:**
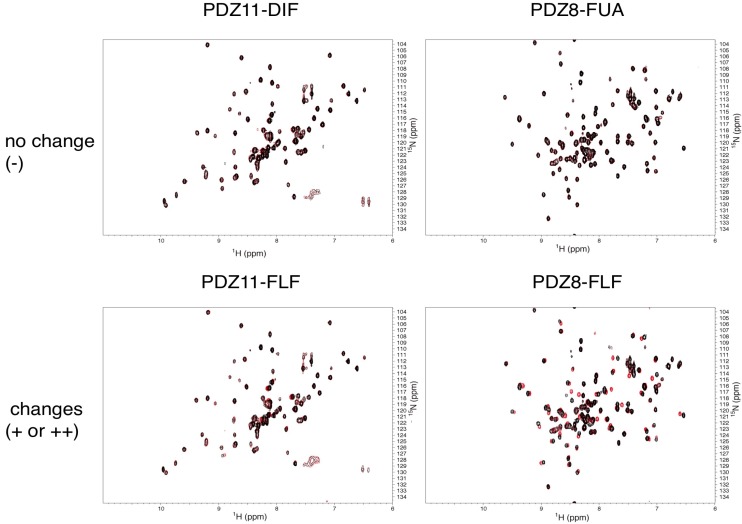
Examples of NMR titration of PDZ domains with each compound. Each overlaid spectrum was derived from a PDZ domain with (red) and without (black) the compound. Upper spectra show results where no signal changes were observed after mixing the PDZ domain with a compound. Lower spectra show the signal changes of the PDZ domain when mixed with a compound.

Next, we focused on three PDZ domains (PDZ7, PDZ8 and PDZ13) that showed the largest chemical shift changes in the presence of any of the cocktails. Additionally, two PDZ domains (PDZ9 and PDZ11) derived from same polypeptide of PDZ13 and PDZ8, were also examined. An additional PDZ domain, the first PDZ domain of mouse ZO1 protein (mZO1-PDZ1), was then used as a positive control with full signal assignment. For these PDZ domains, all 10 individual compounds were titrated under NMR observation. Three of 10 compounds, DIF, FLF, and FUA, bound to four or more PDZ domains (Figure 2, Table 3). All the three compounds showed a fast chemical exchange profile between the bound and the unbound states. Even in the presence of 20 molar equivalents of compounds, the signal changes were not saturated (data not shown). Thus, we assumed that all the binding constants between the compounds and the PDZ domains are as weak as submillimolar range. The compounds DIF (known as diclofenac) and FLF (known as flufenamic acid or flufenac) are both NSAIDs. Compound FUA is fusidic acid, which is used as a bacteriostatic antibiotic. All three compounds possess a carboxylic acid moiety, which fits the C-terminal peptide-binding sites of PDZ domains.

### 2.4. Determination of the Mouse Zo1-PDZ1 Binding Site of the 3 Ligands

As indicated above, three ligands—DIF, FLF, and FUA—were revealed as group-specific binders of PDZ domains with a broader specificity. Therefore, we examined whether these ligands interacted with the first PDZ domain of mouse ZO-1 (mZO1-PDZ1), one of the well-studied canonical PDZ domains [37,38,39]. Ligands (20 molar equivalents) were added to ^15^N-labeled mZO1-PDZ1, and the signal changes were monitored by ^1^H–^15^N SOFAST-HMQC. The three ligands also bound to mZO1-PDZ1, giving 13 signals (DIF), 16 signals (FLF), and 17 signals (FUA) upon binding to the drugs (Table 3). In addition, we determined the dissociation constants (*K_D_*) of DIF and FLF against mZO1-PDZ1 as 1,400 µM and 750 µM, respectively (Supplementary Figure S1). As assumed previously, the interaction between drugs and the PDZ domain was not strong.

**Figure 3:**
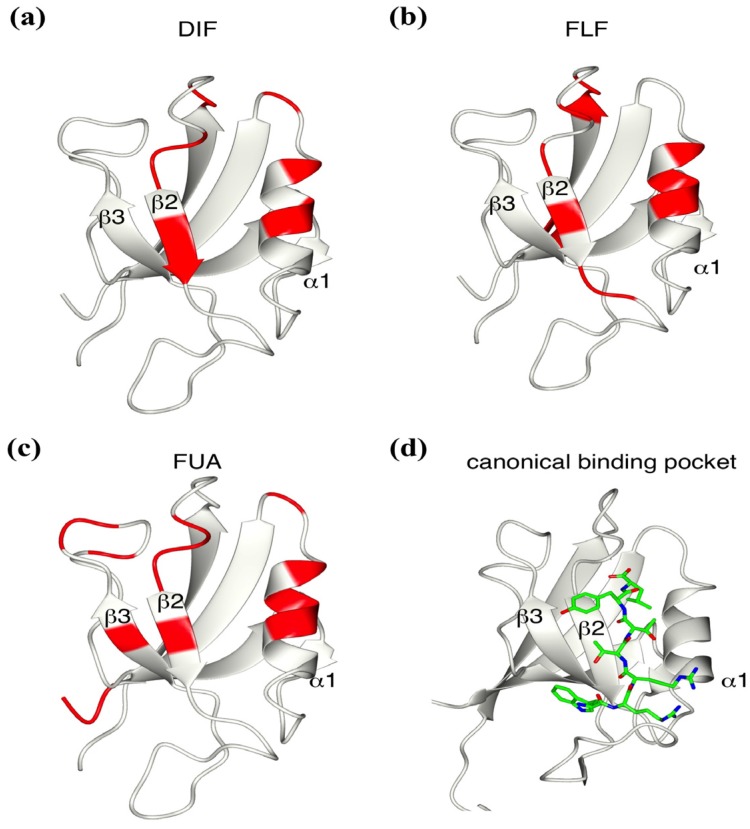
Identification of the interface between the mZO-1 PDZ1 domain and a compound. Mapping of residue signal changes upon mixing with DIF (**a**), FLF (**b**), and FUA (**c**) onto the ribbon model of the mouse ZO-1 PDZ1 domain (PDB:2RRM). (**d**) The ribbon model represents the canonical binding pocket between the PDZ domain and peptide (PDB:2H2B).

Next, we analyzed the NMR signal changes of the residues upon ligand interaction. All residues were colored in red and mapped on to the structure of mZO1-PDZ1 [Figure 3(a–c)]. These residues are located at the loop between β1 and β2 strands (residues 29 and 33–35), the β2 strand (residues 37, and 38), the α1 helix (residues 92, 93, 96, and 97), and the loop between α1 and β6 (residue 99). They surround the canonical ligand binding pocket of the PDZ domains, which recognizes the C-terminal peptide as the physiological PDZ ligand. Considering that all three ligands contain a carboxylate group, mZO1-PDZ1 and the other PDZ domains may recognize these ligands as similar as that the PDZ domains accept their own physiological peptide ligands.

**Table 3:** Summary of the interaction between PDZ domains and small compounds. The number of plus signs indicates the degree of signal changes. The minus sign indicates no signal change. n. e. indicates that the sample was not examined.

ID	A2G	DIF	FUA	SIA	NES	TRP	MPB	FLF	MIL	MNA
PDZ7	−	++	++	−	−	−	−	+	−	−
PDZ8	−	+	−	−	−	−	−	++	−	−
PDZ9	−	+	−	−	−	−	−	+	−	−
PDZ11	−	−	+	−	−	−	−	+	−	−
PDZ13	n. e.	++	+	−	−	−	−	++	−	−
mZO-1 PDZ1	n. e.	+	+	n. e.	n. e.	n. e.	n. e.	+	n. e.	n. e.

FUA affected a larger number of residues compared with DIF, and the residues showing chemical shift changes were widely distributed on one surface of mZO1-PDZ1, probably reflecting a larger molecular size of FUA than that of DIF. In the case of FLF binding, the residues showing changes around the peptide binding pocket are similar to those of DIF, whereas another three residues showed changes on the opposite surface of the binding pocket. It is unclear whether such a difference may reflect a secondary ligand binding site or an allosteric conformational change.

### 2.5. Critical Residues Involved in Accidental PDZ–Diclofenac Interactions

**Figure 4:**
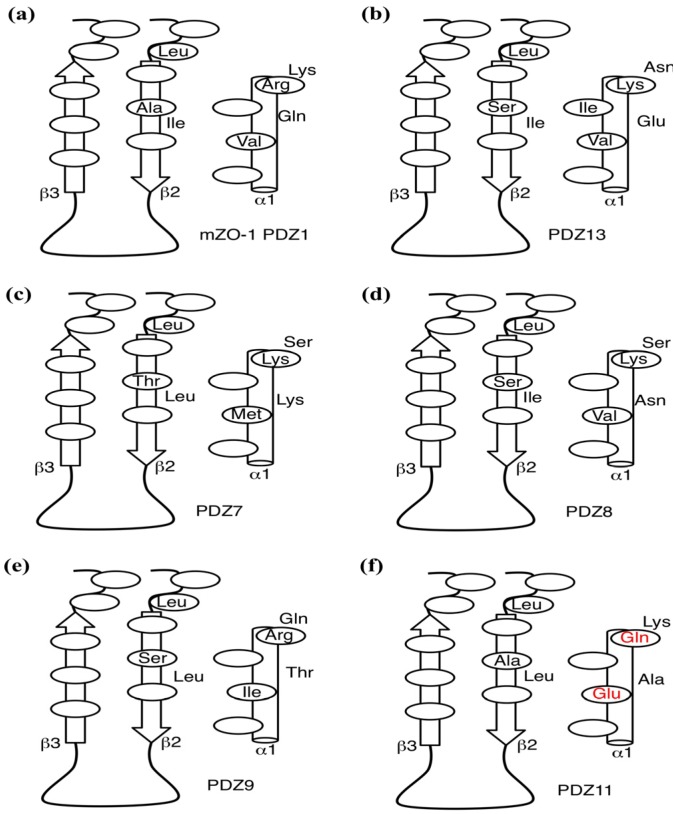
Comparison of the interfacial residues. Schematic of the interfacial residues of mZO-1 PDZ1 (**a**), PDZ13 (**b**), PDZ7 (**c**), PDZ8 (**d**), PDZ9 (**e**), and PDZ11 (**f**) are depicted. The canonical binding pocket lies between a β-sheet composed of β2 and β3 strands and α1 helix, represented as block arrows (β2 and β3 strands) and a cylinder, respectively. Each ellipse represents the location of each residue. The atypical residues of PDZ11 are in red.

We further focused on DIF and FLF because both compounds resemble each other with two common chemical substructures: an amino group cross-links a benzene ring with a carboxylic residue with a benzene ring with halogens. Both drugs bound to most of the PDZs examined; however, only PDZ11 did not bind DIF. We examined the surface residues of the PDZ domains that bound both DIF and FLF. A motif comprising hydrophobic and cationic residues, as V/I/M-X-X-X-R/K, starting at the middle of α1 helix was noted. PDZ11, which did not bind to DIF, has E and Q at the corresponding positions. Moreover, PDZ4 and PDZ10 domains were silent against the water-soluble ligand cocktail and possess K and T, and E and S, respectively (Figure 4). Thus, we hypothesize that the motif V/I/M-X-X-X-R/K is essential for DIF binding of the PDZ domains.

**Figure 5:**
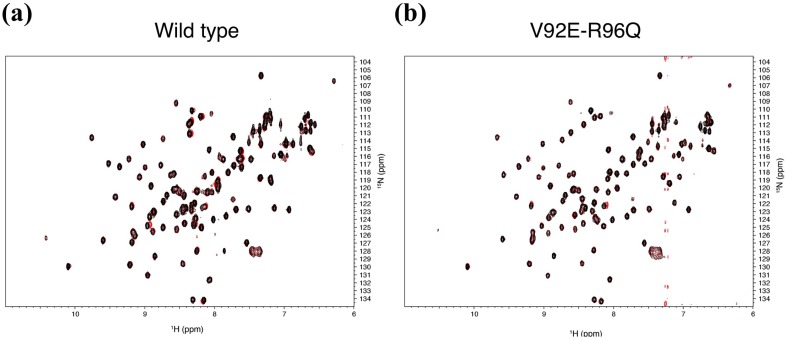
Identification of the key residues of the mZO-1 PDZ1 domain responsible for DIF binding. Overlaid spectra derived from wild type (**a**) and the V92E-R96Q mutant (**b**) of the mZO-1 PDZ1 domain with (red) and without (black) DIF.

In order to confirm the hypothesis, we further constructed a mutant mZO1-PDZ1. The mutant (V92E-R96Q) mZO1-PDZ1 has two residues taken from PDZ11 at the putative DIF-sensitive discrimination site by substituting the corresponding residues of mZO1-PDZ1. ^1^H–^15^N NMR spectra of the mutant showed no significant structural change of the mutant domain compared with that of the wild type, whereas ligand binding with DIF was not detected even in the presence of 20 molar excess of DIF to the PDZ domain (Figure 5). We concluded that the motif V/I/M-X-X-X-R/K may be a good candidate for determining the reactivity of PDZ domains to DIF.

### 2.6. Pharmacological Implications of Accidental PDZ–Diclofenac Interactions

Many research groups are focusing on PDZ domains as the target platform of protein–protein interaction inhibitors [31,32,40,41]. Simultaneously, based on the more physiological interest, genome-wide proteomics studies on all mammalian PDZ domains with their targets were reported [42], indicating the physiological and pharmacological importance of PDZ proteins in the human body. In the current study, we have only showed that DIF and FLF can bind to many of these PDZ domains; however, we did not examine whether DIF or FLF can inhibit the protein–protein interaction of the PDZ domain. A significant structural similarity was observed between DIF, FLF, and the non-peptide Dvl inhibitor 3289-5066 [41] and NHERF1 inhibitors [43]. All of these PDZ inhibitors possess a putative pharmacophore with a benzene ring substituted with a carboxylate moiety. Thus, after several steps of appropriate chemical conversion processes, derivatives of either DIF or FLF may become lead compounds of PDZ inhibitors. Our study on NMR-assisted virtual screening using eF-seek is evaluated as exploratory research in this field.

We demonstrated that the hydrophobic and basic residues on the α1 helix are important in the interaction with DIF. Thus, we searched for PDZ domains having such a pair of residues. Approximately half (138/255) of the PDZ domains were found to contain the pair. Interestingly, all PDZ domains of PSD-93, PSD-95, SAP97, and SAP102, which are scaffold proteins in the synapse, also have the pair, although each protein contains 3 PDZ domains. Moreover, both PDZ1 domains of ZO-2 and ZO-3 involving a formation of a tight junction also have the pair. Thus, many cellular PDZ domains are predicted to interact with DIF.

Although diclofenac is thought to be one of the better-tolerated NSAIDs, several adverse effects have been reported [44,45]. Gastrointestinal complaints, which is the most frequently reported adverse effect, as well as dermatological, cardiovascular [46], hepatic [47], and renal [48] malfunctions should be monitored when diclofenac is prescribed. Although not all of the mechanisms underlying these side effects have been clarified, membrane scaffold proteins harboring the PDZ domains may be a link. In our study, only weak interactions with rapid kinetics between PDZ domains and DIF/FLF were observed. The interactions of these PDZ domains with either DIF or FLF do not readily result in detrimental health effects. Nevertheless, it is possible that other PDZ domains in cells have a high affinity to DIF/FLF and may consequently inhibit their native functions (interaction with targets and sub-cellular localization). Contrary, several PDZ domain proteins are known to play important roles in pain sensation [49,50,51], hyperalgesia [52], and anesthesia [53]. Our results do not rule out that the accidental interaction between DIF/FLF and such the PDZ domain proteins may enhance the anti-inflammatory action of DIF/FLF. All the possibility should be experimentally verified by NMR screening experiments with all PDZ domains in the human genome. Progress of systematic NMR research of PDZ domains may lead to the development of safer NSAIDs.

## 3. Experimental

### 3.1. Preparation of Protein Samples

The expression vectors for the recombinant GST-tagged form of PDZ domains listed in Table 1 were constructed using the PRESAT-vector methodology [33]. Isotopically labeled proteins for NMR titration were generated in *E. coli BL21* (DE3) grown in 1 L M9 minimal medium culture at 20 °C in the presence of [15N]-NH_4_Cl as the sole nitrogen source. The harvested cells were resuspended in lysis buffer (50 mM Tris–HCl, pH 7.5, 150 mM NaCl) and disrupted by sonication. The supernatant was applied to a DEAE–Sepharose (GE Healthcare, Little Chalfont, UK) column and then affinity purified by Glutathione Sepharose 4 Fast Flow (GE Healthcare) chromatography. The GST tag was removed by PreScission protease on beads. The purified proteins were concentrated to ~0.2 mM and dialyzed with 5 mM MES (pH 6.5).

### 3.2. NMR Experiments

NMR experiments were performed on a Bruker Avance III (600 MHz) NMR spectrometer (Bruker, Karlsruhe, Germany) equipped with a cryogenic triple-resonance probe. For the titration study, 25 μM PDZ domain sample was dissolved in 300 μL of 5 mM sodium–MES buffer (pH 6.5), and the ^1^H–^15^N SOFAST-HMQC spectra with and without ligands were measured. In each titration experiment, a final concentration of the compound at 0.5 mM (cocktail or single compound) was added to the proteins. The signal assignment of mZO1-PDZ1 (the first domain of mouse ZO1) has already been published [39]. All NMR spectra were recorded at 288 K. All spectra were processed using NMRPipe [54] and analyzed using SPARKY [55]. All chemical shift changes in the ^1^H–^15^N SOFAST-HMQC spectra were calculated according to the formula {Δδ(^1^H)^2^ + [Δδ(^15^N)/7]^2^}^1/2^. The chemical shift changes were then mapped onto the structure of mZO1-PDZ1 (PDB:2RRM) using OtMG/CCP4mg graphic software [56].

## 4. Conclusions

Three non-peptide PDZ domain ligands, diclofenac, flufenamic acid, and fusidic acid, were found to bind to more than 4 PDZ domains at their canonical peptide binding sites. We examined 10 commercially available compounds, which were selected by genome-wide eF-seek prediction against all PDZ domains in the human genome. This ratio is higher than that of the usual NMR-assisted *in silico* screening, although eF-seek failed to predict some of the newly found PDZ–ligand interactions. Since eF-seek is an evidence-based binding site predictor for only ligands which appear at least once in PDB, eF-seek is not ideal for virtual screening in drug discovery. In contrast, eF-seek is sensitive to shapes and charge distributions of the protein surface, thereby making it useful for comparing proteins in terms of protein–ligand interaction. Finally, with these features, eF-seek is efficient for drug repositioning and/or adverse effect prediction.

## Supplementary Materials

Supplementary materials can be accessed at: http://www.mdpi.com/1420-3049/18/8/9567/s1.
