# Supplementary material for: Accidental Interaction between PDZ Domains and Diclofenac Revealed by NMR-Assisted Virtual Screening
Source: Molecules. 2013 Aug 9;18(8):9567–81. doi: 10.3390/molecules18089567 (PMC6270271; doi:10.3390/molecules18089567)
Supplement: Supplementary file 1 [file molecules-18-09567-s001.pdf]

Supplementary Materials

Figure S1. NMR-binding assay between mZO1-PDZ1 domain and NSAIDs, DIF (a) and FLF (b). Each overlaid spectrum was derived from mZO1-PDZ1 domain with (red) and without (black) drugs. Normalized chemical shift changes for the residues Q84 and G89 and the fitted saturation curves with K_D values of 1,400 µM and 750 µM, respectively, were shown (right).

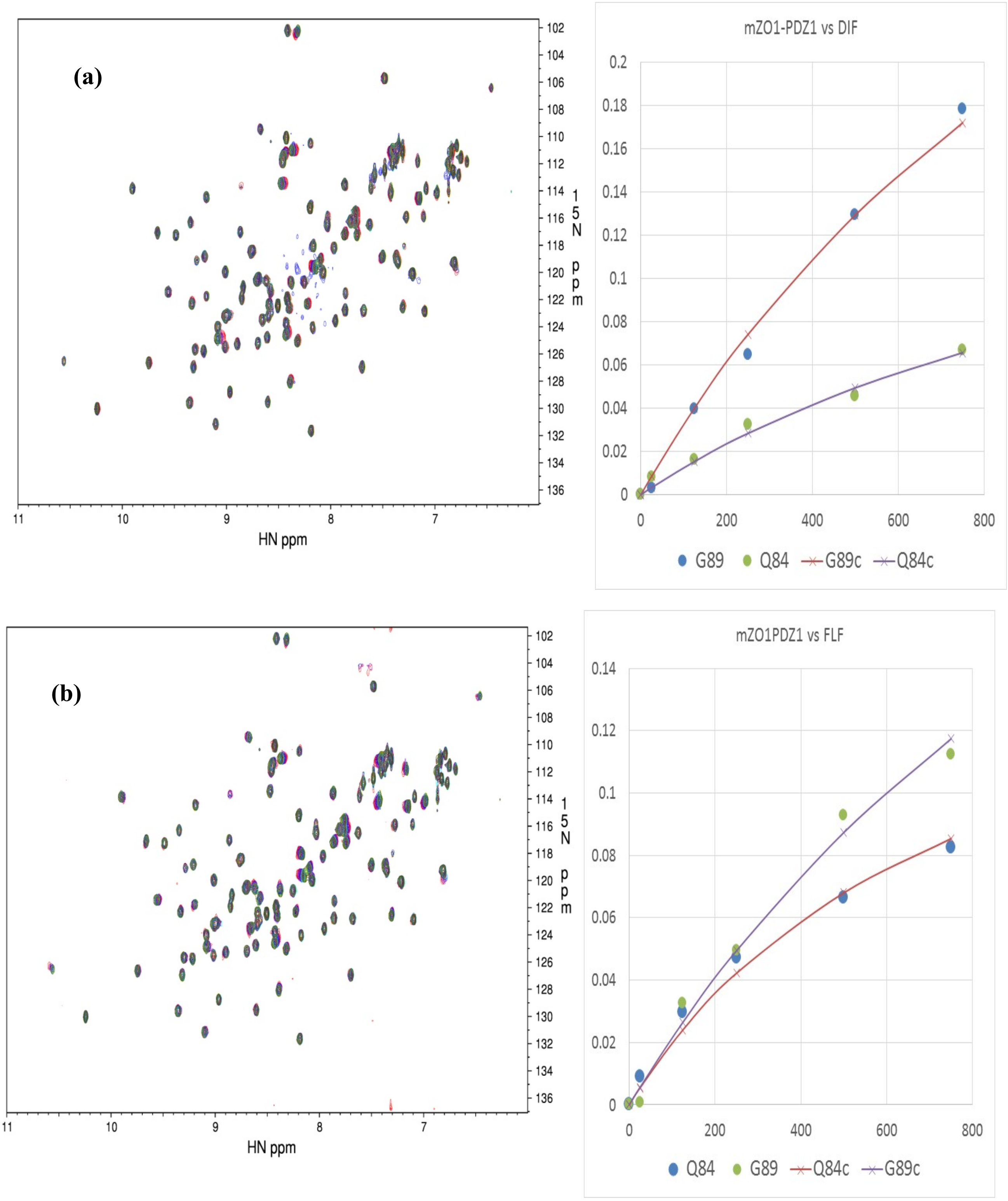

Experimental Section

NMR experiments were performed on a Bruker Avance III (600 MHz) NMR spectrometer (Bruker, Karlsruhe, Germany) equipped with a cryogenic triple-resonance probe. For the titration study, 25 µM mZO1-PDZ1 domain sample was dissolved in 300 µL of 5 mM sodium–MES buffer (pH 6.5), and the ^{1}H–^{15}N SOFAST-HMQC spectra with various concentration (25 µM (1 eq), 125 µM (5 eq), 250 µM (10 eq), 500 µM (20 eq) and 750 (30 eq), respectively), were measured. Signals showing significant chemical shift changes (12 signals for DIF and 11 signals for FLF) were selected, and the normalized chemical shift changes were calculated. Non-linear least square fitting was finally performed to estimate the dissociation constant K_D according to the Equation (1),

$$\delta = \delta_{saturated} \times \frac{([PDZ]_{total} + [L]_{total} + K_D) - sqrt(([PDZ]_{total} + [L]_{total} + K_D)^2 - 4[PDZ]_{total}[L]_{total})}{2[L]_{total}} \quad (1)$$

where δ and $\delta_{saturated}$ are normalized chemical shifts at given ligand concentration and the saturated point, respectively. K_D and $\delta_{saturated}$ values for the selected residues were simultaneously optimized by using SOLVER function in Microsoft Excel software.
